# Psychotherapy and Social Change: Utilizing Principles of Cognitive-Behavioral Therapy to Help Develop New Prejudice-Reduction Interventions

**DOI:** 10.3389/fpsyg.2015.01771

**Published:** 2015-11-20

**Authors:** Michèle D. Birtel, Richard J. Crisp

**Affiliations:** ^1^School of Psychology, University of Surrey, Guildford, UK; ^2^Aston Business School, Aston University, Birmingham, UK

**Keywords:** anxiety, intergroup relations, mental imagery, prejudice, psychotherapy

## Abstract

We propose that key concepts from clinical psychotherapy can inform science-based initiatives aimed at building tolerance and community cohesion. Commonalities in social and clinical psychology are identified regarding (1) distorted thinking (intergroup bias and cognitive bias), (2) stress and coping (at intergroup level and intrapersonal level), and (3) anxiety (intergroup anxiety and pathological anxiety). On this basis we introduce a new cognitive-behavioral model of social change. Mental imagery is the conceptual point of synthesis, and anxiety is at the core, through which new treatment-based approaches to reducing prejudice can be developed. More generally, we argue that this integration is illustrative of broader potential for cross-disciplinary integration in the social and clinical sciences, and has the potential to open up new possibilities and opportunities for both disciplines.

## Introduction

Mental imagery is the experience of “seeing with the mind’s eye or hearing with the mind’s ear” (Kosslyn et al., 2001, p. 635). These images need not to be simply an accurate recall of past events from memory. They can be simulations of future events, a cognitive construction of hypothetical events, or a combination of both (Taylor and Schneider, 1989). A large body of research has demonstrated the benefits of mental imagery in various areas such as health and personality psychology, consumer research, clinical therapy, sports, and intergroup relations (for a review, see Crisp et al., 2011). We provide a new theoretical framework based on mental imagery that integrates research in clinical and social psychology. The aim is to strengthen interventions aimed at promoting tolerance and reducing inequality and discrimination. Practically, through this theoretical integration, and guided by commonalities in conceptual focus, we outline a cognitive-behavioral model of social change (CBM-SC) that locates mental imagery as a “treatment” analog, transposed to the prejudice domain.

Since the Second World War, there has been a growing focus by social researchers on designing and testing psychology-based interventions to reduce prejudice (Watson, 1947). Allport’s (1954) intergroup contact theory is regarded as the cornerstone of theories about how meaningful contact between members of groups with different backgrounds (on the basis of ethnicity, age, sexual orientation, or other dimensions) can reduce prejudice. This notion has been supported by Pettigrew and Tropp’s (2006) meta-analysis of over 500 studies, giving evidence that intergroup contact has a reliable effect in reducing prejudice across different target groups, age groups, contact settings, and geographical areas. Indeed, there is emerging evidence that the concept of contact is even more powerful than previously thought. The notion that individuals do not necessarily have to experience personal contact with outgroup members but can rely on indirect contact experiences has received significant support (Dovidio et al., 2011). Imagined intergroup contact has been proposed as a further implementation of contact theory that can capitalize on the benefits of contact, even where opportunities for contact are unlikely or impossible. It is defined as “the mental simulation of a social interaction with a member or members of an outgroup category” (Crisp and Turner, 2009, p. 234). Miles and Crisp’s (2014) meta-analysis of 70 studies found that simply imagining positive social contact successfully reduces intergroup bias across a broad range of target outgroups and contexts. Previous prejudice-intervention programs designed to change intergroup attitudes, such as the multicultural curricula approach (Appl, 1996) and the anti-racist approach (Dei, 1996), have often only a small impact (Bigler, 1999). Those approaches are typically developed from intuition and creative insight (Aboud and Levy, 2000), rather than research-led theory. Interventions developed from contact theory are based on methods that have been tried and tested in controlled laboratory settings and generate strong and lasting attitude change (Pettigrew, 1998). However, direct contact is sometimes difficult to establish or can have negative consequences (Richeson and Shelton, 2003; Saguy et al., 2009; Vorauer and Sasaki, 2009). Prejudice can be a result of segregation which makes establishing meaningful contact between communities very difficult, with physical manifestations including the Green Line in Cyprus or the West Bank in Israel (Pettigrew, 2008; see also Crisp and Turner, 2009).

**FIGURE 1:**
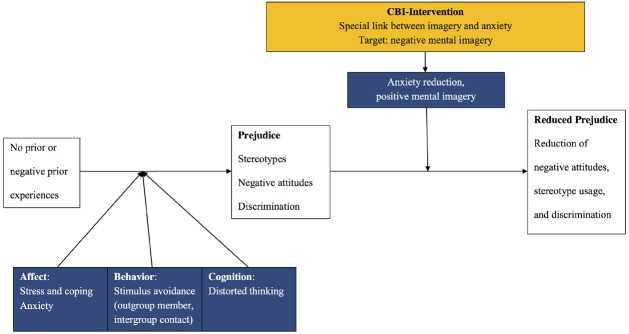
**Cognitive-behavioral model of social change (CBM-SC)**.

As prejudice is a pervasive and critical problem, we need to draw upon a wide range of academic disciplines and perspectives to tackle it. While clinical psychology has been using methods to treat negative affect, cognition, and behavior for a long time, the principles underlying such techniques have largely been ignored as possible sources of theoretical inspiration by social scientists outside clinical psychology. In the following, we outline a psychotherapy inspired CBM-SC (see Figure 1).

## The ABC of Prejudice and Mental Health

Prejudice is a negative attitude toward a group and its individual members because of their group membership (Brown, 2011). Based on the multicomponent model of attitudes (Zanna and Rempel, 1988), prejudice is the combination of negative affect (feelings, A), derogatory cognitive beliefs (stereotypes, C), and hostile behavior (discrimination, B; Brown, 2011). Affective prejudice expresses itself in negative emotions toward the outgroup, i.e., what individuals dislike about the outgroup. Cognitive prejudice expresses itself in beliefs about the personal attributes of a group of people. Behavioral prejudice expresses itself in negative behaviors toward the outgroup (Farley, 2005).

Prejudice often refers to social groups such as ethnic minorities, but prejudice in terms of stigma can also be employed to people suffering from mental illness. Prejudice can lead to contact avoidance, and negative verbal and non-verbal behavior toward the stigmatized group (Stephan and Stephan, 1985). Negative or no prior experiences with outgroup members have consequences for all components of the ABC of prejudice—affect, cognition, and behavior. Individuals who do not have much contact with outgroup members experience negative affect in terms of stress or even physiological distress at the prospect of intergroup contact (e.g., Blascovich et al., 2001; Trawalter et al., 2009). A cognitive consequence of lacking contact is the formation and maintenance of social stereotypes. As a result, individuals either display hostile behavior toward the outgroup, or avoid outgroup contact (Stephan and Stephan, 1985; Plant and Devine, 2003).

Processes of prejudice, stereotypes, discrimination, or social identity threat have consequences for disadvantaged group members’ health (for a review, see Major et al., 2013). Health inequalities between members of socially disadvantaged and advantaged groups are a pervasive, cross-cultural problem. Experiences of social devaluation based on group membership leads to health inequalities by affecting three areas: (1) stress, (2) health behaviors, and (3) healthcare interactions. Firstly, stigmatized groups experience discrimination which leads to enhanced life stress that negatively affects their health. Secondly, being target of prejudice triggers unhealthy behaviors and coping strategies (e.g., smoking, drugs, comfort eating) that have negative effects on health. Thirdly, social biases can negatively affect the quality and nature of the healthcare that members of disadvantaged groups receive (e.g., time spent with patient, appropriate treatment, miscommunication; Major et al., 2013). Improving affect, cognition, and behavior related to prejudice via a cognitive-behavioral intergroup (CBI) intervention not only aims at reducing social disharmony and intergroup conflict. It has a much broader aim of reducing discrimination and the health inequalities and costs that come hand in hand with it.

Similar to the ABC of prejudice, there is a vicious cycle in mental illness, and similar mechanisms have been identified to cause mental health problems, which cognitive-behavioral therapy (CBT) targets. A negative event (e.g., encountering a rude person) can lead to irrational beliefs and distorted cognitions (e.g., “I’m unlovable”) that in turn can evoke negative affective (e.g., feeling sad) and behavioral (e.g., withdrawal) consequences. How people think, how they feel, and how they act all influence each other, resulting in a vicious cognitive triangle of affect, cognition and behavior (Ellis, 1957, 1962; Beck, 1967). CBT breaks this cycle of negative thoughts, feelings, and, behaviors, helping patients to reinterpret the negative activating events (Rothbaum et al., 2000). CBT has established a method that could be employed to mitigate prejudice.

We propose that a prejudice-intervention that adapts principles used in CBT could be helpful in reducing prejudice by changing negative affect, stereotypes, and discrimination. Some recent work in the intergroup relations domain has started to link principles from clinical psychology and prejudice. Analogies and commonalities in social and clinical psychology can be found regarding (1) *distorted thinking* (Cox et al., 2012), (2) *stress and coping* (Trawalter et al., 2009), and (3) *anxiety* (Birtel and Crisp, 2012b). Previous research primarily focused on single aspects of the ABC, for example Cox et al. (2012) focused on cognition, Birtel and Crisp (2012b) focused on affect, and Trawalter et al. (2009) focused on stress reactions and coping behavior (the B of prejudice).

We review these three areas of research and discuss how this existing literature supports a CBT-inspired approach to understanding and reducing prejudice. We expand on previous models by proposing a model that integrates these programs to inform the development of social-clinical interventions for reducing prejudice. We further propose that mental imagery is the common underlying dimension connecting these three domains of research, and that emotion, in particular anxiety, is at the core.

### Distorted Thinking (C)

Distorted thinking, referring to the cognition of the ABC of mental states, occurs both in intergroup relations (e.g., intergroup bias) and mental illness (e.g., cognitive bias in depression; see point 1 in Table 1).

**TABLE 1:** **Examples of commonalities in prejudice and mental health disorders concepts**.

**Commonality**	**Prejudice**	**Mental health disorders**
***Cognition (C)***		
1. Distorted thinking (Cox et al., 2012)	Intergroup bias (Hewstone et al., 2002)	Cognitive bias in depression (Beck, 2008)
***Behavior (B)***		
2a. Stress	Primary appraisal: intergroup stress, e.g., anxiety, physiological threat (Blascovich et al., 2001; Stephan and Stephan, 1985)	Transactional model of stress and coping (Lazarus and Folkman, 1984)
2b. Coping (Trawalter et al., 2009)	Antagonism, avoidance, freezing, and positive engagement	Attack, avoid, inactivity, and positive actions
***Affect (A)***		
3. Anxiety (Birtel and Crisp, 2012b)	Intergroup anxiety leading to contact avoidance or negative behavior toward outgroup (Stephan and Stephan, 1985; Pettigrew and Tropp, 2008)	Pathological anxiety leading to phobic stimulus avoidance or negative behavior toward self (National Institute of Mental Health [NIMH], 2013)
4. Comorbidity	Deprejudice: comorbid prejudice and depression (Cox et al., 2012)	Comorbid anxiety disorder and depression (Clark and Watson, 1991; Hirschfeld, 2001)
	Key mediator anxiety: comorbid prejudice and anxiety (Stephan and Stephan, 1985; Pettigrew and Tropp, 2008)	
5. Special link between mental imagery and anxiety	Improves attitudes, intentions, self-efficacy and behavior within and outside intergroup context (Crisp et al., 2011)	Key role in developing, maintaining and treating anxiety disorders (Holmes and Mathews, 2005)
6. Mental imagery based intervention	Imagined contact (Crisp and Turner, 2012)	Depression: cognitive bias modification intervention (Lang et al., 2012)
	Exposure therapy approach of imagined contact (Birtel and Crisp, 2012b)	Anxiety disorder: exposure therapy (Foa et al., 1991)

In an intergroup context, individuals are biased toward their own group (*intergroup bias*). This means that ingroup members are generally evaluated more positively than outgroup members. Intergroup bias can take the form of favoring one’s ingroup over the outgroup (i.e., ingroup favoritism), or, less common, derogating the outgroup (i.e., outgroup derogation). The bias is displayed through negative attitudes (prejudice), negative cognitions (stereotypes), and negative behavior (discrimination; Hewstone et al., 2002). Intergroup bias satisfies people’s need for a positive social identity (Tajfel and Turner, 1979) by enhancing self-esteem (Abrams and Hogg, 1990; Hewstone et al., 2002).

In a clinical context, according to Beck’s (1967) cognitive model of depression, distorted thinking is characteristic for psychological disorders (Beck, 2008). *Cognitive biases* in information-processing, as well as dysfunctional attitudes and beliefs are risk factors of depression (Beck, 2008). Similar to depressed patients, who show a systematic negative cognitive bias in attention and recall (Beck, 1999, 2008), prejudiced individuals show a systematic negative bias when it comes to recalling intergroup encounters (West et al., 2011; Experiments 1 and 2). However, when individuals are explicitly instructed to think of a positive intergroup encounter, the quality of the imagined encounter is higher, and even improves intergroup attitudes (West et al., 2011; Experiment 3).

Cox et al. (2012) proposed the deprejudice quadruplex as the link between prejudice and depression. They integrate perspectives on prejudice and depression, with the term “deprejudice” describing comorbid depression and prejudice. At the core of Cox et al.’s (2012) deprejudice quadruplex is the assumption that stereotypes about others in prejudiced individuals, and schemas about the self in depressed individuals share the same type of cognitive structures which are automatically activated. Cognitive structures, stereotypes and schemas share several characteristics: They have an affective component and consequences for behavior, and they lead to cognitive biases which are rather resistant to change. Whereas depressed individuals are generally motivated to reduce their own depression (self-directed prejudice), not all prejudiced individuals are motivated to enhance positive attitudes toward others (other-directed prejudice).

A reason to believe that prejudice-interventions based on clinical models could be useful is the subjectivity and malleability of social reality. Previous research supports the notion that distorted thinking is a subjectively distorted view of reality. Stereotypes can result in distorted thinking. Stereotypes are beliefs about the personal attributes of a group of people and used to simplify the world, to categorize people into social groups on the basis of ethnicity, gender or other common attributes. Although stereotypes can be accurate (Jussim et al., 2009), they create bias because they are routinely applied to all members of a group. For example, all older adults could be seen as incompetent and slow (e.g., Cuddy et al., 2005). Stereotypes become a problem when they are overgeneralized, inaccurate, and resistant to new information (Devine, 1989), which in turn can propagate prejudice. Interestingly, while prejudice models have always implicitly assumed this, they have not linked this to clinical models (for a more in-depth elaboration, see Cox et al., 2012). In other words, social reality is subjectively construed just like the perception of reality that leads to distorted beliefs. If social psychologists view stereotypes as a form of distorted thinking, similar to mental health models, then accepting that *social reality* is as *subjectively malleable* as the development of (for example) phobic beliefs may also mean that we can adapt principles from CBT to help correct this distorted reality.

### Stress and Coping (B)

Stress responses and the associated coping behavior not only occur at an intrapersonal level (e.g., personal stress), but also at an intergroup level (e.g., intergroup stress; see points 2a and 2b in Table 1).

At the intergroup level, social interactions with outgroup members are a source of threat and therefore cause stress (e.g., Blascovich et al., 2001; Trawalter et al., 2009), leaving individuals to decide on coping behavior to deal with the stress, the B of the ABC. Individuals interacting with stigmatized group members experience discomfort in form of uncertainty, anxiety, and even threat (Blascovich et al., 2001). Blascovich et al. (2001) suggest a biopsychosocial approach to stigma. They found threat responses to an interaction with various stigmatized partners on subjective, physiological, and behavioral measures.

At an intrapersonal level, stress is an aversive psychological state and individuals are motivated to reduce it. According to Lazarus and Folkman’s (1984) transactional model of stress and coping, individuals who perceive a potential stressor as stressful (primary appraisal), evaluate their resources as either exceeding the demands of the stressor (challenge) or as insufficient (threat). Depending on this secondary appraisal, individuals choose an appropriate coping strategy to manage stress (e.g., avoid the stressor or positive actions, Lazarus and Folkman, 1984).

Trawalter et al. (2009) applied Lazarus and Folkman’s (1984) stress and coping model to the intergroup context. In their adapted framework, they perceive intergroup contact as stressor, and reconceptualize behavior in intergroup interactions as stress reactions and coping responses. They emphasize the importance of cognitive appraisals of a potential stressor as threat and of a person’s resources in shaping coping behavior. Prejudice and uncertainty linked to interracial contact often lead both majority and minority group members to appraise interracial interactions as threat instead of challenge, which in turn leads to affective, physiological, cognitive, and behavioral stress reactions (Shelton et al., 2005). According to Shelton (2003), majority and minority groups differ in their interpersonal concerns. Minority groups are concerned with being the target of discrimination and engage in behavioral strategies to ensure a positive interaction (Shelton et al., 2005). Majority groups feel concerned about appearing prejudiced and experience intergroup anxiety (Stephan and Stephan, 1985) or even threat in terms of values and economy (Stephan and Stephan, 2000).

### Anxiety (A)

Anxiety, the affective component of the ABC, is not only linked to prejudice and intergroup stress (e.g., intergroup anxiety), but also to anxiety disorders and depression (e.g., pathological anxiety; see point 3 in Table 1).

Looking at intergroup relations, anxiety at the prospect of intergroup contact can lead to stress. Negative expectations of rejection or discrimination during cross-group interactions or because of fears that the interaction partner, or the respondents themselves, may behave in an incompetent or offensive manner can arouse *intergroup anxiety* as a form of intergroup stress. Anxiety about potentially poor, embarrassing or difficult interactions with stigmatized group members inhibits interest in cross-group contact, and even can lead to hostility (Stephan and Stephan, 1985; Plant and Devine, 2003). It leads individuals to avoid contact (Stephan and Stephan, 1985), compels them to rely on stereotypes (Wilder, 1993), it lowers the communication quality (Gudykunst and Shapiro, 1996), and leads individuals to experience physiological stress when such interactions occur (Blascovich et al., 2001). Intergroup anxiety plays a key role in intergroup relations. Reduced anxiety is the primary mechanism through which exposure (i.e., contact) reduces prejudice (Pettigrew and Tropp, 2006, 2008), and much work has shown anxiety to be a major mediator in prejudice reduction (e.g., Blascovich et al., 2001; Voci and Hewstone, 2003; Page-Gould et al., 2008).

Research developing interventions to reduce prejudice have correspondingly focused on combating anxiety about interacting with stigmatized groups. Most notable amongst these approaches is intergroup contact theory (Allport, 1954) which is regarded as most influential for improving intergroup relations between conflicting ethnic groups through meaningful contact between those members compared to merely living side-by-side. Contact reduces prejudice by building affective ties (reducing intergroup anxiety, enhancing empathy) or through cognitive processes such as creating common social identities emphasizing shared membership (Pettigrew, 1998).

Looking at clinical psychology, depressive disorders and anxiety disorders often co-occur (Hirschfeld, 2001), and over half of patients with major depression have comorbid depression and anxiety (see point 4 in Table 1), especially anxiety symptoms such as worry, psychic and somatic anxiety (Hirschfeld, 2001; Aina and Susman, 2006). According to Clark and Watson’s (1991) tripartite model of anxiety and depression, anxiety and depression are highly correlated due to a common underlying distress factor which they name negative affectivity (NA). Individuals high in NA are easily distressed, anxious and have negative views of themselves. Negative attributional style as a consequence of high NA is therefore not unique to depression but also common in anxiety (Clark et al., 1994). When normal anxiety exceeds itself in duration, intensity and frequency, it becomes a problem, impairing people’s day-to-day functioning, leading to an anxiety disorder (e.g., post-traumatic stress disorder PTSD, social phobia, or specific phobias such as animals or height). Characteristic of *pathological anxiety* is unrealistic fear and worry as a response to a stressor. Extreme anxiety leads people to avoid certain situations (National Institute of Mental Health [NIMH], 2013), similarly to individuals avoiding situations in which they meet certain outgroup members.

### Summary

In summary, intergroup interactions can be stressful and lead to cognitive biases because of the anxiety involved in those uncertain contexts, which can lead to stimulus avoidance (e.g., contact situation or outgroup member; Stephan and Stephan, 1985) and cognitive depletion (Richeson and Trawalter, 2005; Richeson and Shelton, 2007).

So far, we have demonstrated links between social and clinical psychological intervention perspectives in terms of affect, cognition, and behavior. The research reviewed above supports the view that emotion is at the core. First, distorted thinking in form of intergroup bias and cognitive bias in depression both lead to negative emotions in terms of anxiety or hostility in intergroup contexts, and low mood in depression. Second, perceived stress in intergroup relations as well as everyday life can be linked to uncertainty, anxiety and threat. Third, anxiety is not only a key mediator in the contact-prejudice relationship but also a key factor in maintaining anxiety disorders.

Going beyond integrating the discussed three models, we next discuss how mental imagery can be powerful not only as a psychotherapeutic technique to tackle negative emotions (and in particular anxiety), but also to mitigate prejudice. For example, distorted cognitive beliefs in mental health disorders are malleable and can be changed by mental imagery which in turn changes emotions. This further supports the use of prejudice-interventions that are based on clinical models.

## A Special Link Between Mental Imagery and Anxiety

A large body of research has demonstrated the benefits of mental imagery in various areas such as health and personality psychology, consumer research, clinical therapy, and sports. Imagery improves attitudes, intentions, self-efficacy and behavior (for a review, see Crisp et al., 2011; see point 5 in Table 1). A common, disorder-maintaining symptom in anxiety disorders is negative imagery (Hirsch and Holmes, 2007). Research in clinical and cognitive psychology proposes a special link between mental imagery and emotion, especially anxiety (Kosslyn, 1994; Holmes and Mathews, 2005; see point 5 in Table 1). Imagery has a more powerful effect on emotions like anxiety than verbal processing (Holmes and Mathews, 2005; Holmes et al., 2008a,b), and even prevents negative mood more effectively than verbal thinking (“cognitive vaccine,” Holmes et al., 2009). Mental imagery influences emotions in both positive and negative ways (Holmes and Mathews, 2010). In Holmes and Mathews (2005), participants received descriptions of unpleasant scenarios. One half imagined these events, the other half thought about their verbal meaning. Participants in the imagery condition experienced a greater increase in anxiety compared to participants in the verbal condition. Mental imagery not only induces greater negative affect (Holmes and Mathews, 2005), but also greater positive affect (Holmes et al., 2006), than verbal processing.

Research on social phobia has emphasized how negative imagery can be detrimental for social interactions. Social phobia is a form of anxiety that occurs in social situations (Hirsch and Holmes, 2007). Individuals fear interacting with other people and being negatively evaluated by them, especially in unfamiliar situations. As a result, individuals tend to avoid these situations. Self-imagery influences anxiety and behavior in both individuals high and low in social anxiety. The negative self-imagery of people high in social anxiety led to anxiety and reduced the quality of a conversation with another person (e.g., conversational flow, interestingness of conversation). Creating a non-negative self-imagery of being relaxed in a social situation in people with social anxiety reduced anxiety, and led to a better performance rated by a conversational partner (Hirsch et al., 2004). Participants low in social anxiety who adopted a negative self-image prior to giving a speech reported greater anxiety and showed lower performance compared to participants who adopted a positive self-imagery (Hirsch et al., 2006). The authors conclude that negative imagery plays a causal role in developing and maintaining social anxiety.

### Mental Imagery Interventions

Mental imagery has been the target of interventions in (1) clinical psychology, e.g., depression, emotional disorders (e.g., Foa et al., 1991; Lang et al., 2012; see point 6 in Table 1); and (2) social psychology, e.g., prejudice, performance (for a review, see Crisp et al., 2011; see point 6 in Table 1).

Mental imagery and the reduction of negative affect is the point of synthesis between previous models. There is extensive evidence that mental imagery is beneficial in many areas of psychology as well as in the domain of prejudice, for example in the treatment of anxiety disorders (Holmes and Mathews, 2005), but also in prejudice-interventions to reduce intergroup anxiety (Crisp and Turner, 2012). Adopting principles of exposure therapy, targeting the A of prejudice, in this case anxiety, through mental imagery, is hypothesized to not only reduce negative affect but also change distorted thinking, for example in terms of beliefs about future contact. Changing affect and cognition then should result in behavior change, for example less discrimination (Birtel and Crisp, 2012b). Targeting anxiety is important in both social and clinical psychology, as it is a key emotion in both prejudice and mental illness. Both anxiety disorders and prejudice are maintained by anxiety which leads to avoidance of the anxiety-provoking stimulus. Cognitive representations of intergroup contact contain negative responses to outgroup members in terms of affect, cognition, and behavior (e.g., Blascovich et al., 2001; Riek et al., 2006; Gonsalkorale et al., 2007), analog to cognitive representations of a phobic stimulus (e.g., Hope et al., 1990; Rapee and Heimberg, 1997).

In clinical psychology, imagery has been powerful in reducing negative affect and cognition. Looking at research on depression, Lang et al. (2012) showed, for the first time, that a 1-week computerized cognitive bias modification intervention (CBM-I) that targets interpretation bias in depression via positive mental imagery significantly reduced depressive symptoms, cognitive bias, and intrusive symptoms even 2 weeks after the intervention compared to a control group. Results from a large randomized controlled trial show that a mental imagery based cognitive training can improve anhedonia in people with depression (Blackwell et al., 2015). Looking at research on emotional disorders, given the special link between mental imagery and anxiety, clinical treatments of anxiety disorders focus on repeating or modifying such images with the aim of reducing their emotional power. Early forms of treatment used imagery as part of a desensitization approach for treating phobias (Wolpe, 1958), while more recent forms use CBT (Hirsch et al., 2003).

Anxiety disorders are maintained by avoidance. In their emotional-processing theory, Foa and Kozak (1986) argue that fear emerges through a development of a fear memory which elicits escape and avoidance. Their logic is based on Lang’s 1977, 1979) bio-informational theory of emotional imagery in which fear represents a network in memory—the “fear structure.” These cognitive representations contain stimulus information, responses to the stimulus (verbal, physiological, and behavioral), and interpretive information about meaning (threat or danger). Thus, according to emotional-processing theory (Foa and Kozak, 1986; Foa and McNally, 1996) successful exposure therapies reduce phobias and anxiety disorders if two conditions are met. First, the fear memory needs to be activated in order to be modified. Second, corrective learning must take place, i.e., corrective information incompatible with the fear structure must be available and integrated. Result is a non-fear structure that either replaces or competes with the old, anxiety-provoking structure. Craske et al. (2008) argue that an index of corrective learning is not the fear levels but fear toleration. Instead of focusing on fear reduction as the mechanism, they consider inhibitory processes as central and reconceptualize exposure therapy as reshaping memory, developing non-threat associations and retrieving those new associations over time and context.

There are a number of forms of CBT that draw upon the power of imagery in tackling anxiety disorders. Exposure therapy (e.g., systematic desensitization, imaginal exposure or *in vivo* exposure; Foa et al., 1991) confronts the patient with fear-evoking objects or situations within a safe environment, with patients instructed to actively visualize and describe the phobic stimulus. Similarly, in systematic desensitization, therapists work with the client to form a graduated anxiety hierarchy and to tackle these with concomitant imaginal relaxation techniques, as these are antagonist to an anxious physiological state. This technique has been found to be a highly effective way to reduce anxiety in clinical contexts (Wolpe, 1958; Tarrier et al., 1999; Rothbaum and Schwartz, 2002; Choy et al., 2007; Himle, 2007).

In social psychology, the power of mental imagery seems to extend to the domain of prejudice. Mental imagery plays an important role in social situations as well as in intergroup perceptions and interactions. A mental experience of a particular social context can have the same effect as an actual experience of that context (Blair et al., 2001; Garcia et al., 2002). For example, Garcia et al. (2002) showed that activating the psychological construct of a group of people led to an implicit bystander effect. Participants who mentally simulated having a meal with 10 people were less willing to help the experimenter in a second study compared with participants who imagined having a meal with only one person. Among the few strategies to moderate and control implicit stereotypes that have shown to be effective, Blair et al. (2001) used a new strategy based on mental imagery. Throughout five experiments, implicit stereotypes were weaker after having engaged in counterstereotypic mental imagery (e.g., a strong woman) compared to participants who engaged in neutral (e.g., vacation in Caribbean), stereotypic or no mental imagery. Crisp and Turner (2009, 2012) proposed that mentally simulating a positive social interaction with a person from another group capitalizes on the extended psychological benefits of the intergroup contact concept (“imagined contact hypothesis”). Mentally simulating positive social contact has established positive effects on attitudes, intentions, self-efficacy and behavior [i.e., factors of Ajzen and Madden’s (1986) theory planned behavior] toward various target groups in terms of ethnicity, religion, sexual orientation, age or mental health (Miles and Crisp, 2014).

Mental simulation of social contact could be especially useful for people high in intergroup anxiety (Birtel and Crisp, 2012a), and there is already established evidence that it is effective in reducing intergroup anxiety (e.g., Turner et al., 2007). Birtel and Crisp (2012b) tested whether adapting the principle of pre-positive *negative* imaginal exposure would enhance the effectiveness of subsequent positive imaginal exposure. The results of three experiments, targeting a range of stigmatized groups (adults with schizophrenia, gay men, and British Muslims), showed that compared to purely positive interventions, imagining a single negative encounter, just prior to a positive imaginal intervention, resulted in significantly reduced prejudice. Furthermore, reduced anxiety uniquely derived from the mixed-valence imagery task statistically explained enhanced intentions to engage positively with the previously stigmatized group in the future. When it comes to promoting positive group perceptions, negativity is not all bad; and a small dose, administered just prior to a positively-focused intervention, can be surprisingly effective in reducing prejudice toward stigmatized groups.

### Summary

Summarizing the review of clinical therapies, imagery and anxiety: The concepts of prejudice and mental health disorders seem to have more commonalities than researchers have been aware of (Trawalter et al., 2009; Birtel and Crisp, 2012b; Cox et al., 2012). Stereotypes in the social psychology literature and schemas in the clinical psychology literature can lead to *distorted thinking* such as intergroup bias and cognitive bias in depression. *Anxiety* inhibits and maintains negative behavior in both social and clinical contexts such as avoiding the outgroup or phobic stimulus (e.g., spider, social situation). Using the transactional model of *stress and coping* (Lazarus and Folkman, 1984), intergroup interactions can lead to stress and behavior in intergroup interactions are a coping response. Similar to *comorbid* anxiety disorder and depression (Clark and Watson, 1991; Hirschfeld, 2001), prejudice often comes hand in hand with anxiety as the key mediator between contact and prejudice (Pettigrew and Tropp, 2008), or with depression (deprejudice; Cox et al., 2012).

Mental imagery is powerful in reducing negative affect and cognitive bias in mental health problems (Holmes and Mathews, 2005; Lang et al., 2012) and intergroup relations (Crisp and Turner, 2012) which interventions should make use of. CBT inspired prejudice-reduction interventions could target both cognitions and affect in order to lead to behavior change.

## Cognitive-Behavioral Intergroup Intervention

The reviewed literature suggests that we need to target the ABC of prejudice when developing prejudice-interventions. Specifically, we need to (a) improve negative affect (e.g., anxiety), (b) change distorted thinking (derogatory beliefs, stereotypes), and reduce hostile behavior (discrimination), all of them are consequence of negative past experiences (e.g., prior contact with the outgroup). We have drawn analogies between social and clinical psychology with respect to the ABC and provided an integration of previous models: Intergroup anxiety can be considered as a form of non-pathological anxiety (Birtel and Crisp, 2012b), intergroup bias can be considered as a form of cognitive bias in depression (see also deprejudice quadruplex, Cox et al., 2012), and intergroup interactions are a source of stress that lead to various coping behaviors (Trawalter et al., 2009).

Several explanations are currently offered for the exposure mechanism, e.g., habituation, counterconditioning, or more cognitive processes like cognitive restructuring, self-efficacy or emotional processing models (for a review, see Tryon, 2005). Being exposed to the anxiety-provoking stimulus (e.g., mental image of a spider or an outgroup member) within a safe environment is expected to trigger objective thinking instead of dysfunctional thinking, and to enhance self-efficacy in coping with the anxiety.

With our central message, that principles underlying psychotherapy interventions utilizing mental imagery could inspire new prejudice-reduction interventions, we aim to stimulate future research into the development of new, efficacious methods to promoting positive social change. Examples for psychotherapy-informed prejudice-interventions (e.g., in schools or in the workplace) could be (a) a mental simulation intervention based on exposure therapy, (b) a cognitive-behavioral prejudice-reduction program based on negative and positive mental imagery and cognitions, or (c) a mindfulness meditation program based on negative and positive mental imagery. While there is only preliminary evidence for the effectiveness of negative-then-positive imaginal exposure in the laboratory (see Birtel and Crisp, 2012b), future research could examine whether repeated exposure to a negative mental imagery followed by a positive mental imagery is feasible in the laboratory and the field. Cognitive-behavioral interventions that combine findings related to negative cognitions and negative emotions could be tested to see whether they can successfully reduce affective and cognitive forms of prejudice, and whether these changes translate into discriminatory behavior. Mindfulness meditation programs, running over a period of time, could be designed with and without reference to the outgroup, making use of mental imagery that is negative (namely the “difficult person” stage during loving-kindness meditation), neutral (the “stranger” condition) or positive (the “friend” condition; Kang et al., 2014; Parks et al., 2014). While our review focused on anxiety as the primary mediator between intergroup contact and prejudice, a mindfulness meditation intervention could test both mediators, anxiety and empathy, given that mindfulness meditation aims at enhancing empathy in individuals. Furthermore, future research should examine the role of other emotions relevant to prejudice, such as empathy, trust, disgust or anger (see, for example intergroup emotions theory, e.g., Mackie et al., 2000).

Critics of mental imagery interventions for prejudice-reduction have argued that these interventions may be insufficient to tackle prejudice outside the laboratory (e.g., Bigler and Hughes, 2010; Lee and Jussim, 2010). While we agree the problem of prejudice cannot be solved by focusing only on individual cognition in the laboratory, we also believe it cannot be solved without it either. The next step is to adapt a mental imagery intervention tested in the laboratory to a practical method that can be used in the field by practitioners and policy-makers for the use in education and organizations (e.g., Crisp and Turner, 2010). Although research has shown that the positive effect of a 3-week intervention on prejudice can at least last a week (Vezzali et al., 2012), prejudice-interventions based on mental imagery may not be expected to be not as long-lasting as interventions based on direct contact. Therefore, an intervention in the field would go beyond a single, brief exercise, and involve multiple sessions to achieve sustainable changes in people’s prejudice and discriminatory behavior (e.g., Crisp and Turner, 2009).

## Discussion and Conclusion

Negative thoughts, feelings and beliefs are the basis for prejudice toward all manner of groups in societies across the world (Paolini et al., 2010). Given the importance of negative affect—in particular anxiety—for reducing prejudice, it makes sense to develop interventions that specifically target anxiety as the root cause of prejudice. We here propose that principles used in the psychotherapeutic treatment of depression and anxiety disorders can help inform new “cognitive-behavioral” interventions to help tackle the problem of prejudice against stigmatized groups.

We have drawn analogies and commonalities between social and clinical psychology in terms of (1) distorted thinking (Cox et al., 2012), (2) stress and coping (Trawalter et al., 2009), and (3) anxiety (Birtel and Crisp, 2012b). On the basis of this integrative analysis, we propose a cognitive-behavioral model that uses the power of mental imagery to promote positive social change. Specifically, we aimed to integrate research findings and methods of social and clinical psychology to design a new class of prejudice-reduction intervention. Taking an established social psychological concept—social contact—and reconceptualizing it in a way that it unites the field with another discipline within psychology, namely the large literature on CBT, could open new possibilities and opportunities in reducing social conflict.

Furthermore, while this review discussed research that focuses on the social consequences of prejudice, there is a great need for future research to examine whether prejudice-interventions based on clinical psychological principles could be effective in reducing the negative health consequences of prejudice. Promoting good mental health and wellbeing (through reduced prejudice), which is more than simply the absence of mental illness, has not only health but also social and economic benefits for single individuals as well as whole communities (Department of Health, 2011). Good mental health not only leads to better physical health and lower mortality, but also to greater educational achievement and higher work performance, as well as less crime and improved community life. Therefore, tackling the problem of anti-social behavior and prejudice not only reduces crime but also improves mental health and the stigma related with it.

Finally, as this CBI approach to social change is fairly new, key questions remain that are yet unanswered: Are CBT-informed interventions feasible and acceptable in real-conflict settings? To which contexts can CBT-informed interventions be applied? What are the risks of using a CBT-informed approach? These are questions which only future research can answer, and help to ascertain whether such interventions have scientific, social and public health relevance.

### Conflict of Interest Statement

The authors declare that the research was conducted in the absence of any commercial or financial relationships that could be construed as a potential conflict of interest.
